# Attitudes to Drug Policy in Australia, 2001–2022/23: What Does This Mean for Drug Policy Reform in Australia?

**DOI:** 10.1111/dar.70130

**Published:** 2026-03-15

**Authors:** Zachary Lloyd, Amanda Roxburgh

**Affiliations:** ^1^ Harm and Risk Reduction Burnet Institute Melbourne Australia; ^2^ Monash Addiction Research Centre Monash University Melbourne Australia; ^3^ National Drug and Alcohol Research Centre, UNSW Sydney Sydney Australia

**Keywords:** decriminalisation, drug policy reform, harm reduction, legalisation, public opinion

## Abstract

**Introduction:**

We are at a critical juncture for drug policy reform. Toxic drug supply is driving unprecedented deaths in North America, with countries calling for decriminalisation. Policy reform in Australia has occurred in some states (e.g., decriminalisation in the Australian Capital Territory), stalling in others (e.g., the second supervised injecting facility [SIF] in Victoria). The objectives of this paper are to present trends in attitudes to drug policy in Australia, 2001–2022/23 and media reporting of related policies, to explore how public opinion and media attention have changed over time.

**Methods:**

Analysis of (i) National Drug Strategy Household Survey data on attitudes to drugs/drug policies; and (ii) Factiva for media reporting.

**Results:**

In 2022/23, Australians supported needle and syringe programs (67.8%), SIFs (58.3%), take home naloxone (60.9%) and drug checking services (64.4%). They opposed legalisation of methamphetamine (86.6%), heroin (85.6%), cocaine (78.2%) and ecstasy (75.8%), while a larger proportion supported cannabis legalisation (44.6%). Australians supported a health response to possession of drugs (57.3% for methamphetamine, 80.9% for cannabis) rather than a criminal response. Methamphetamine was increasingly reported as the drug of most concern (2.9%–42.5%), decreasing for heroin (44.9%–11.4%), 2001–2022/23. Methamphetamine media articles increased (272–2426); heroin decreased (2136–973), 2000–2023. Mentions increased for SIFs (spiking at 175, 2019) and decriminalisation/legalisation (spiking at 242, 2019).

**Discussion and Conclusions:**

Support for harm reduction remains high and low for legalisation. Media focused on methamphetamine, SIFs and decriminalisation/legalisation. Despite opposition towards legalisation, most Australians favoured a health response to drug use.

## Key Point

- Australians are largely supportive of harm reduction initiatives.
- Support for the legalisation of heroin, methamphetamine, cocaine and ecstasy is low.
- Australians, however, predominantly prefer a health response for possession of illicit substances.
- Media counts were highest for methamphetamine, injecting rooms and decriminalisation.

## 1 Introduction

We are at a critical juncture for drug policy reform internationally, with toxic drug supply presenting challenges in many regions globally [1]. North America continues to experience an unprecedented overdose crisis, largely driven by fentanyl analogues [2] and more recently other synthetic opioids such as nitazene compounds [3]. While Australia has not experienced levels of harm seen in North America, clusters of fentanyl analogue and novel synthetic opioid (nitazene, U447700) poisonings [4] and fatalities [5, 6] have been reported. Additionally, harms related to established illicit drugs such as crack cocaine and methamphetamine are also increasing in certain regions including Australia [7, 8, 9]. These trends present significant challenges for drug‐related harms and drug policy reform internationally [10].

A range of harm reduction responses have been implemented internationally, including: (i) drug consumption rooms (supervised injecting facilities—SIFs, or overdose prevention sites); (ii) drug checking services; (iii) take home naloxone (THN); and (iv) safe supply distribution. There is strong evidence that drug consumption rooms [11, 12, 13, 14], drug checking services [15, 16] and THN programs [17] reduce drug‐related harms and these services are available in many countries [18]. Safe supply programs have primarily been implemented in Canada, with an emergent evidence base [19, 20, 21, 22]. There have also been increasing calls for drug decriminalisation and in some settings, legalisation. In 2019, the United Nations endorsed decriminalisation of drug use and possession [23]; as of 2017 approximately 23 countries had implemented decriminalisation or legalisation of previously illicit drugs. The evidence for these policies currently remains limited, with the exception of cannabis; a recent US systematic review highlighted complications in evaluating the impacts of both policies due to substantial heterogeneity in policy design [23].

The rate at which harm reduction strategies are being implemented is not keeping pace with the significant drug‐related harms occurring in certain parts of the world [24, 25, 26]. Drug policy reform is often difficult to achieve and, in theory, is influenced by multiple factors: socio‐political ideology [27, 28]; media portrayals of drugs and people who use drugs [27, 29, 30, 31]; empirical research evidence [27] and a mechanism to present this evidence to government [32] and public opinion [27, 33, 34]. In reality, media narratives and socio‐political ideology frequently dominate drug policy reform in Australia [27, 31, 35].

Historically, Australia pioneered drug policy reform, implementing needle and syringe programs (NSP) nationally in the 1980s to curb the spread of HIV [36] and establishing the first SIF in the English‐speaking world in the 2000s [37]. These reforms were largely reactive, implemented in response to a perceived crisis rather than strategically planned [37, 38]. Drug policy reform in Australia compared to some countries (e.g., Canada) has slowed. While some states have made progress (e.g., decriminalisation in the Australian Capital Territory [39] and drug checking in the Australian Capital Territory, Victoria and New South Wales [40, 41]), reforms have stalled in others (e.g., second SIF abandoned in Victoria) [42]. This paper presents two key indicators influencing drug policy reform: (i) population‐based public opinion; and (ii) media reporting.

Previous Australian research on attitudes to drug policy has demonstrated associations between media reporting, stigma and drug‐related attitudes, including the under reporting of methamphetamine use by the general population [43, 44]. Studies have also documented growing support in Australia for legalising cannabis, ecstasy, and cocaine for personal use [45] and also for harm reduction initiatives such as NSPs, supervised injectable opioid treatment (SIOT) [46] and SIFs [47]. Importantly, one of the consistent correlates of support across this literature is previous use or personal experiences related to illicit drugs [45, 47, 48, 49].

Our paper extends this work by looking at attitudes over a longer time period (2001–2022/23) and across a broader range of drug policies and drug law reform items using nationally representative survey data. We also extend reporting by including those who are ‘ambivalent’ (i.e., neither support or oppose) towards drug policies in our analyses, as there is strong evidence these people may change their minds [50]. Finally, we present more recent trends in media reporting of harm reduction policies and drug law reform given the media influence documented in previous research [43].

## 2 Methods

### 2.1 Data Sources

#### 2.1.1 National Drug Strategy Household Survey (NDSHS)

This study utilised data from eight waves (2001–2022/23) of the NDSHS. The NDSHS is a population survey that has been conducted every 3 years since 1985 in all states and territories of Australia, and forms part of the Australian government's National Drug Strategy. The NDSHS collects data on Australians' use of, and perceptions related to alcohol, tobacco and other illicit drugs. It also collects data on attitudes towards harm reduction interventions such as SIFs, NSPs and THN. Data are used to inform service delivery and policy reform within Australia.

Respondents are selected through a complex, multi‐stage stratified randomised process designed to be representative of the Australian population. The NDSHS excludes sampling of individuals who live in non‐private residences (e.g., hotels, shelters and rough sleepers) and institutionalised settings (hospitals, rehabilitation centres, etc.).

Response rates have remained stable (43.9%–53.0%), while sample sizes (21,663–29,445) and the survey modes offered have changed over the years. The survey methodology has been described in greater detail elsewhere [51].

#### 2.1.2 Factiva

Searches of 16 major Australian newspapers (print and online versions—Table 1) were conducted using Factiva with dates restricted to January 2000–December 2023. A range of key terms were utilised to pick up media mentions of substances that represented ‘a drug problem’ such as methamphetamine and heroin. Searches were also conducted for media mentions of specific drug policies such as a ‘trial of prescribed heroin’, ‘take‐home naloxone’ or ‘supervised injecting facilities’. Media searches were conducted to identify references to the decriminalisation and legalisation of illicit drugs.

**TABLE 1:** List of major newspapers searched in Factiva.

Newspaper name	National or jurisdictional
*The Advertiser*	Jurisdictional
*The Age*	Jurisdictional
*The Australian*	National
*Australian Financial Review*	National
*Canberra Times*	Jurisdictional
*Courier Mail*	Jurisdictional
*Daily Telegraph*	Jurisdictional
*Herald Sun*	Jurisdictional
*Hobart Mercury*	Jurisdictional
*Northern Territory News*	Jurisdictional
*Sunday Age*	Jurisdictional
*Sydney Morning Herald*	Jurisdictional
*Sunday Herald Sun*	Jurisdictional
*Sunday Times*	Jurisdictional
*Weekend Australian*	National
*The West Australian*	Jurisdictional

Search terms were kept broad to pick up any media mentions of the topics, except for methamphetamine. Searches related to methamphetamine media mentions were conducted broadly initially to pick up any reference and then again using negative qualifying terms such as ‘ice epidemic’, ‘methamphetamine epidemic’ and ‘methamphetamine scourge’.

### 2.2 Ethics

Ethics approval for this study was obtained from The Alfred Human Research Ethics Committee (study: 47/20) and approval to access the NDSHS data was granted by the Australian Institute of Health and Welfare.

### 2.3 Measures

Attitudes towards NSPs, SIFs, drug checking services, a trial of prescribed heroin (i.e., SIOT) and THN, and the legalisation of certain illicit drugs (e.g., heroin, methamphetamine, cocaine, ecstasy, marijuana) for personal use were analysed. Responses were collected using a Likert‐type item with the following options: strongly support, support, neither support nor oppose, oppose, strongly oppose. Although respondents were also able to select ‘don't know enough to say’, data were not available for these responses in all years, so we excluded them from analyses. Previous research has demonstrated that individuals who report ‘Don't know enough to say’ differ from those who report ambivalence, and indeed, these two response options represent substantively different stances [47]. While this research demonstrated that over time trends remain broadly the same with and without their inclusion, it should be noted exclusion of these responses has the potential to conflate the overall reported proportions. The outcome measure was collapsed to: ‘support’ (strongly support and support), ‘ambivalent’ (neither support nor oppose) and ‘oppose’ (strongly oppose and oppose).

The questions related to harm reduction policies and legalisation have changed over time (outlined in Tables A1 and A2, respectively). Notably, ecstasy was included in the list of drugs for legalisation from 2007 onwards. Take‐home naloxone was added as a drug policy option in 2016, and a separate question on drug checking services was added in 2019.

Respondents are also asked (2010–2022/23) ‘When people talk about a “drug problem” which is the first drug you think of?’. Between 2001 and 2007, respondents were asked ‘When people talk about a “drug problem” which are the first two drugs you think of?’ The first response was included for all years.

To understand attitudes to different response options to possession of illicit drugs for personal use, we analysed the questions ‘What single action best describes what you think should happen to anyone found in possession of small quantities of the following drugs for personal use (cannabis, ecstasy, heroin, methamphetamine)?’ and ‘Do you think the possession of small quantities of Marijuana/Cannabis for personal non‐medical use should be a criminal offence, that is, should offenders get a criminal record?’. The first question was categorised as follows: a health response (including ‘no action’, ‘warning only’, ‘referral to drug education’, ‘referral to treatment’); a civil response (‘something similar to a parking fine, up to $200’, ‘a substantial fine, around $1000’, ‘a community service order’) and a criminal response (‘weekend detention’, ‘a prison sentence’). The second question was a yes–no response.

### 2.4 Analysis

Weighted proportions and 95% confidence intervals were calculated to present changes in Australian attitudes towards drug policies (NSPs, SIFs, legalisation of illicit drugs, etc.) over time. To assess statistical changes in opinions over time, several ordinal logistic regression models were fitted with year included as a continuous exposure and age, sex and recent (past 12 month) illicit drug use modelled as covariates. For attitudes towards legalisation of individual drug types, recent use of the respective drug (e.g., cannabis use for cannabis legalisation) was included in each model. The outcome measure was modelled as 0 support, 1 ambivalent and 2 oppose. We tested the proportional odds assumption with the Wald test to determine whether the effects of the covariates on the cumulative odds of being in a higher or lower category remains constant across different levels of the outcome measure [52]. For example, in a model adjusting for survey year, age, sex and recent drug use, we found the proportional odds assumption was violated for attitudes towards SIFs, meaning the effect of survey year, age, sex and recent drug use, was not consistent across the levels of the outcome measure (support, ambivalent, oppose). A partial proportional odds model was therefore used [52], resulting in two odds ratios for oppose versus support and oppose versus ambivalent. This was the case for all ordinal regression models.

Logistic regression was used to assess changes in proportions reporting the most appropriate response to possession of illicit drugs for personal use (2001–2022/23). Adjusted odds ratios, 95% confidence intervals and *p*‐values are reported. All model statistics are presented in Tables A3, A4, A5.

Analyses were conducted in Stata V17 using the *svyset* feature and the appropriate weighting strata and cluster variables. Missing data were excluded from reporting, resulting in complete case analysis. Raw counts of media mention of specific harm reduction policies (e.g., SIFs, NSPs), decriminalisation and legalisation, and mentions of methamphetamine‐ and heroin‐related articles are presented for the years 2000–2023.

## 3 Results

### 3.1 Changes in Attitudes Towards Harm Reduction Initiatives (2001–2022/23)

**FIGURE 1:**
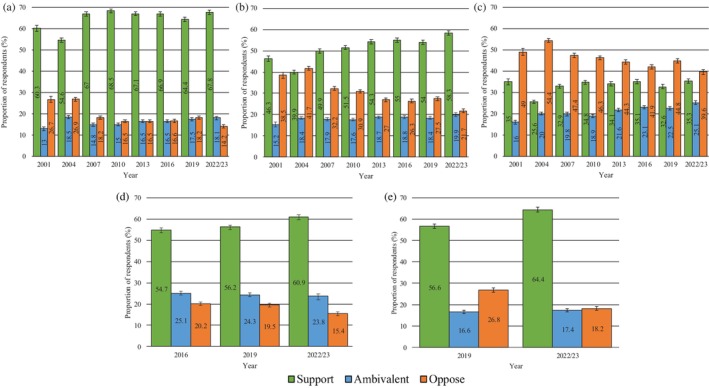
Changes in attitudes towards harm reduction initiatives (2001–2022/23). (a) Needle syringe programs, (b) supervised injecting facilities, (c) trial of prescribed heroin, (d) take‐home naloxone and (e) drug checking services. *Note:* Data presented are weighted. Error bars represent 95% confidence intervals.

In 2022/23, Australians largely supported NSPs (67.8%), SIFs (58.3%), THN (60.9%) and drug checking services (64.4%) (Figure 1). In contrast, findings were mixed regarding a trial of prescribed heroin (35.3% support; 39.6% oppose) (Figure 1c). Analysis of trends over time shows that when holding age, sex and drug use constant, the odds of opposing versus supporting or being ambivalent across all harm reduction policies decreased significantly over time (between 2% and 6% decrease for every unit increase in year) (Figure 1, Table A3).

### 3.2 Changes in Attitudes Towards the Legalisation of Substances for Personal Use (2001–2022/23)

**FIGURE 2:**
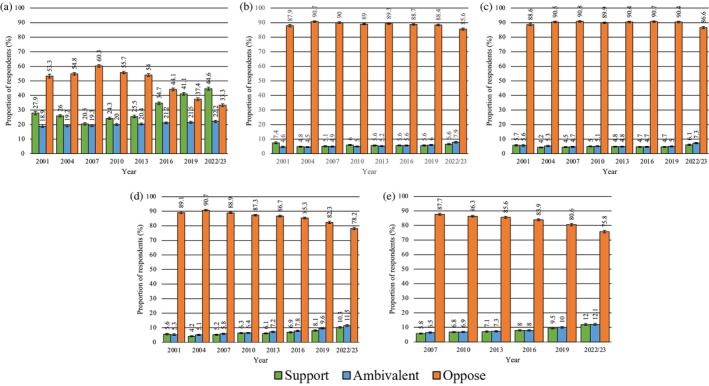
Changes in attitudes towards the legalisation of illicit substances for personal use (2001–2022/23). (a) Cannabis, (b) heroin, (c) methamphetamine, (d) cocaine and (e) ecstasy. *Note:* Data presented are weighted. Error bars represent 95% confidence intervals.

In 2022/23 attitudes towards the legalisation of cannabis for personal use were largely supportive (44.6% of Australians) (Figure 2a). Smaller proportions in 2022/23 were supportive of legalisation of heroin, methamphetamine, cocaine and ecstasy for personal use (ranging from 6.1% for methamphetamine to 12% for ecstasy) (Figure 2b–e). Analysis of trends over time, however, shows that the odds of opposing versus supporting or being ambivalent towards the legalisation of cannabis, methamphetamine, heroin and cocaine have decreased significantly (Figure 2; Table A4). However, the magnitude of these changes is modest, with reductions of only 1%–2% per year.

### 3.3 Changes in Respondent's Perception of What Substance Constitutes the ‘Drug Problem’

**FIGURE 3:**
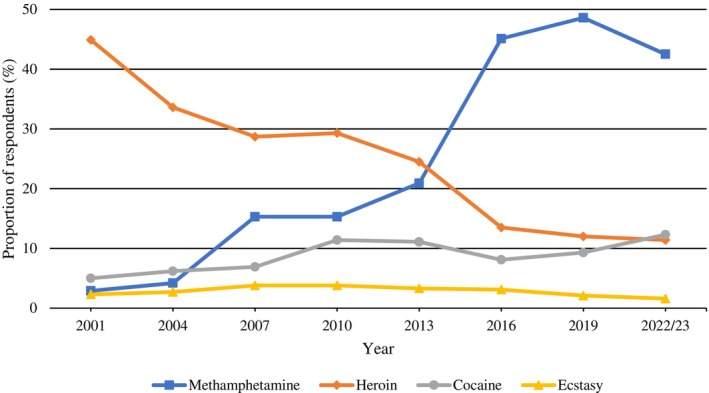
Changes in proportions nominating which substance represents the ‘drug problem’ of most concern (2001–2022/23). *Note:* Data presented are weighted.

There was an overall increase in the proportion of respondents nominating methamphetamine as the first drug they think of when people talk about a ‘drug problem’ (2001: 2.9%; 2022/23: 42.5%; Figure 3) with parallel decreases in proportions nominating heroin (2001: 44.9%; 2022/23: 11.4%). Perceptions that cocaine represented the ‘drug problem’ increased from 5% to 11.4% (2001–2010), remaining relatively unchanged through to 2022/23. Perceptions that ecstasy constituted the ‘drug problem’ have remained low and stable over the years (2001: 2.3%; 2022/23: 1.6%).

### 3.4 Changes in Attitudes to Towards a Range of Responses to Possession of Illicit Drugs

**FIGURE 4:**
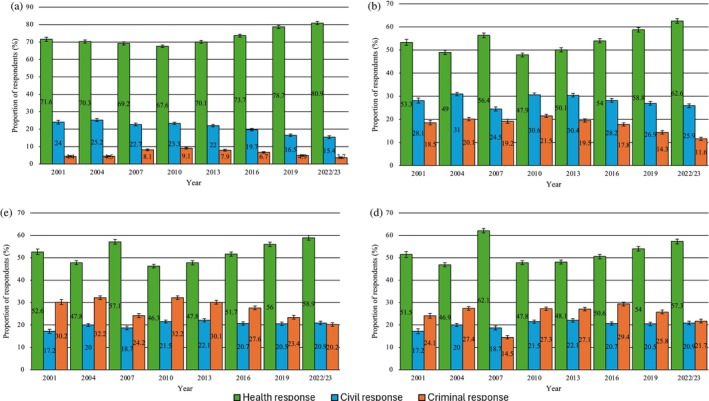
Changes in attitudes towards different responses to the possession of illicit drugs (2001–2022/23). (a) Cannabis, (b) ecstasy, (c) heroin and (d) methamphetamine. *Note:* Data presented are weighted. Error bars represent 95% confidence intervals.

**FIGURE 5:**
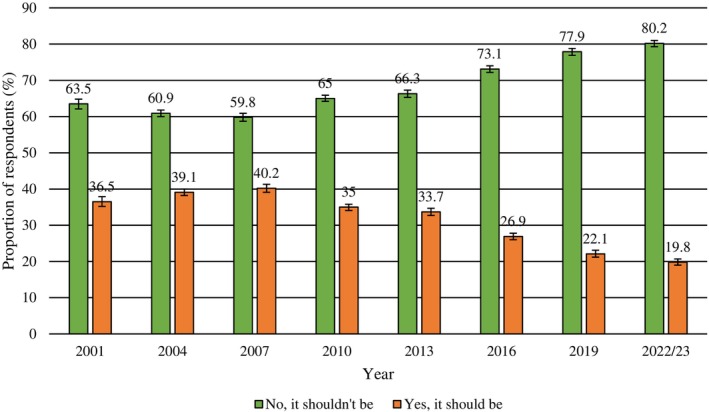
Changes in attitudes towards cannabis possession being a criminal offence (2001–2022/23). *Note:* Data presented are weighted. Error bars represent 95% confidence intervals.

The majority of Australians supported a health response to possession across all four drugs, with smaller proportions supporting civil and criminal responses (Figure 4). There was a significant increase (2001–2022/23) in the odds of respondents supporting a health response to possession for personal use across all illicit drugs, although changes for heroin and methamphetamine were relatively small (Figure 4; Table A5). Comparatively, there was a significant decrease in the proportion of respondents who supported a criminal response. This decline was notably small for cannabis (with recorded proportions remaining lower than 10% across the study period) and for methamphetamine (recorded proportions supporting a criminal response were higher at around one in five people across the study period). There was a significant decrease in proportions supporting a civil response to cannabis and methamphetamine possession; for cannabis this appeared to occur in parallel with increases in support for a health response, and for methamphetamine, a mix of increasing support for a health response and fluctuations in support for a criminal response. Increasing proportions of Australians believe cannabis possession should not be a criminal offence (63.5%, 2001; 80.2%, 2022/23) (Figure 5).

### 3.5 Media Mentions

#### 3.5.1 Trends in Media Reporting of Methamphetamine and Heroin

**FIGURE 6:**
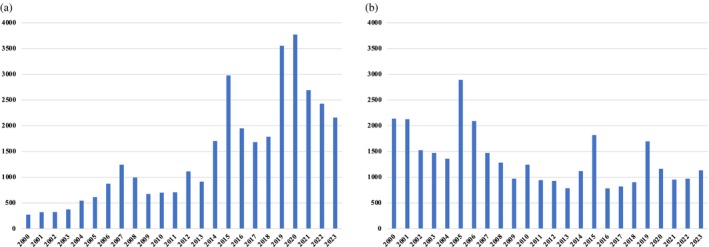
Trends in Australian newspapers reporting on methamphetamine and heroin (2000–2023). (a) Media mentions of methamphetamine and (b) media mentions of heroin. *Note:* Data presented are raw counts.

Media mentions for methamphetamine have gradually increased over time, with notable spikes in 2007 (*n* = 1244), 2015 (*n* = 2979), 2019 (*n* = 3555) and 2020 (*n* = 3700; Figure 6a). Although mentions decreased after 2020, they were 185% higher in 2023 than in 2000. Comparatively, media mentions of heroin decreased overall by 88% between 2000 and 2023, despite substantial spikes in 2005 (*n* = 2890), 2015 (*n* = 1819) and 2019 (*n* = 1695) (Figure 6b). In 2023, media mentions of methamphetamine were almost double those of heroin.

#### 3.5.2 Trends in Media Reporting of Harm Reduction Initiatives

**FIGURE 7:**
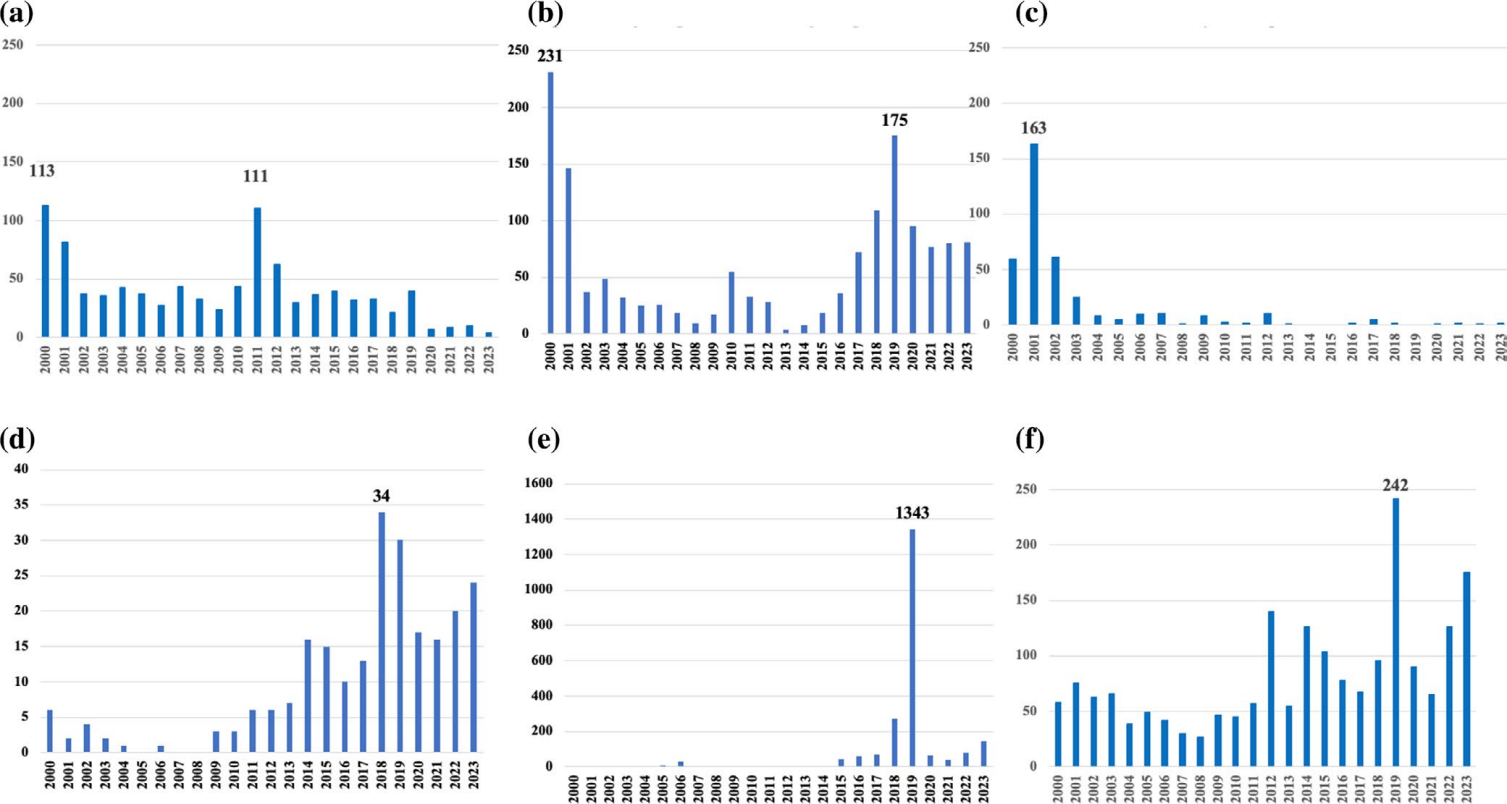
Trends in Australian newspapers reporting on drug policies (2000–2023). (a) Count of articles containing one of the following terms: needle and syringe program OR needle exchange OR needle exchange program. (b) Count of articles containing one of the following terms: supervised injecting centre OR drug consumption room OR supervised injecting room OR safe injecting room. (c) Count of articles containing one of the following terms: heroin trial OR heroin assisted treatment OR prescribed heroin treatment OR hydromorphone trial. (d) Count of articles containing one of the following terms: take‐home naloxone OR take home naloxone OR naloxone. (e) Count of articles containing one of the following terms: pill testing OR drug checking OR drug testing OR pill‐testing OR drug‐checking OR drug‐testing. (f) Count of articles containing one of the following terms: drug AND decriminalisation OR drug AND legalisation. *Note:* Data presented are raw counts.

Media reporting on NSPs remained relatively low between 2000 and 2023 except for 2000 and 2011 when there were over 100 articles reported (Figure 7a). SIF media reporting has fluctuated however, there was a spike in 2000 (*N* = 231), the year preceding the opening of the first service, the Medically Supervised Injecting Centre in Sydney and again in 2019 (*N* = 175), the year following the opening of the Medically Supervised Injecting Room (MSIR) in Melbourne. SIF media articles remained higher in the latter years 2017–2023 (Figure 7b). Media on prescribed heroin treatment/hydromorphone trials remained very low except for 163 articles published in 2001 (Figure 7c). Articles on THN steadily increased between 2016 and 2023 but remain much lower than for other harm reductions initiatives (Figure 7d). Drug checking articles have fluctuated, with a comparatively large spike (relative to other initiatives) occurring in 2019 (*N* = 1343; Figure 7e). Articles on decriminalisation/legalisation have been more consistently featured (comparative to the other initiatives) in the media between 2000 and 2023, spiking to 242 in 2019 (Figure 7f).

## 4 Discussion

Australians in this study were largely supportive of harm reduction interventions (including NSPs, SIFs, THN and drug checking services), with support increasing over time. Previous research on attitudes suggests growing support may be indicative of people who are more directly affected by drug issues, or more knowledgeable about these interventions [34, 53, 54]. However, attitudes towards drug policy are complex and shaped by multiple factors (e.g., socioeconomic status, political ideology, exposure to media reporting and experience of drug use) [43, 55, 56].

We saw a concurrent rise in ambivalence towards several initiatives (notably for SIFs and a prescribed heroin trial). Canadian research, exploring community attitudes towards SIFs, found that ambivalence was linked to a limited understanding of SIFs and the proposed benefits [57]. While not measured in the current study, it offers one possible explanation for ambivalence among our respondents. Persistent misconceptions about harm reduction services, such as concerns about encouraging drug use or worsening public amenity, may also contribute [37]. Systematic reviews and evaluations of SIFs and NSPs do not support these misconceptions, demonstrating improved public amenity and public order outcomes in the areas these services are operating [12, 13, 58, 59, 60].

Attitudes towards a trial of prescribed heroin (i.e., SIOT) were more mixed than for other harm reduction initiatives. Although SIOT has a long history of policy discussion in Australia, commencing as early as the 1980's, public exposure has been limited [61]. Legislation preventing the importation of medical grade heroin (diamorphine) into Australia is still in place [62]; however, the first trial of SIOT in Australia, utilising hydromorphone as an alternative, was conducted in Sydney in 2022–2024 [63]. A second trial, announced in 2024 for Melbourne is yet to commence [64]. Media reporting in the current study showed very little discussion of SIOT during the latter time‐period, which may, in part, be why public opinion remains mixed. Asking about ‘prescribed heroin’ may also be misleading as it does not convey that this initiative represents evidence‐based treatment, nor does it convey that it involves pharmaceutical opioids (i.e., medical‐grade heroin—diamorphine or hydromorphone).

Support for the legalisation of illicit drugs (except cannabis) remains low. Notably, opposition towards the legalisation of methamphetamine is high. Media coverage of methamphetamine increased during the study period. This reporting is often alarmist, using stigmatising language such as ‘meth crisis’ [65] or ‘ice epidemic’ [66], while typically focusing on the crime and legal consequences of methamphetamine use [67]. Earlier research demonstrates an association between increased media attention on methamphetamine and an underreporting of methamphetamine use among the general population and that media portrayals of illicit drugs significantly influence attitudes [68]. The media's portrayal of illicit drugs in Australia has potentially played a significant role in shaping the public's perceptions of drug policy reform and ultimately, drug policy reform itself [44, 69, 70]. As one theorist posits ‘the media both reflects and creates the opinions of members of the public’ [71]. Our findings showed that methamphetamine has become increasingly viewed as a ‘problem drug’ by Australians, correlating with the relatively high opposition to its legalisation, and the increasing media attention demonstrated in the current study.

Despite opposition to legalisation, more than half of Australians support a health rather than a criminal response to possession of cannabis, ecstasy, methamphetamine and heroin. Findings across these questions suggest attitudes are more nuanced than either support for legalisation or for criminalisation.

Media coverage of SIFs spiked in 2019 coinciding with the opening of the MSIR in Melbourne in July 2019 [72]. AOD Media Watch, a group of expert clinicians and researchers, published several articles on the sustained negative media coverage of the Melbourne MSIR in Victoria over the past 5 years [35, 73, 74]. Indeed, language used (e.g., ‘injecting room fury’, ‘rejecting room’ and ‘junkie town’) suggests wide‐spread public opposition to these services [73, 74, 75] which is not supported by the current study or by earlier research [47]. This negative coverage may have influenced the VIC Government's April 2024 decision to abandon plans for a second service in Melbourne's CBD [35, 76]. Portrayals in the media of SIFs and other harm reduction initiatives often do not reflect the evidence supporting their effectiveness, leading to policy decisions that may not align with effective public health responses [35, 77].

### 4.1 Implications

Changes in drug markets occurring globally, and in Australia (particularly, the emergence of the potent synthetic opioids) [78, 79, 80], underscore the need for more proactive drug policy reform [81]. Expansion of already established and well‐supported harm reduction strategies such as upscaling of THN and broader implementation of SIFs and drug checking services across the country may help mitigate harms associated with increasingly volatile drug supplies [79, 81]. While public opinion rarely directly influences policy reform, the current study demonstrates strong support for harm reduction initiatives; accordingly, public opinion should not be presented as a barrier to their further expansion in Australia [47].

The increasing (albeit minimal) ambivalence towards harm reduction services highlights the potential value of clear public education campaigns about these initiatives and their associated evidence base. An important example of this is the evidence hub established by the Drug Policy Modelling Program at the University of New South Wales ahead of the 2024 NSW Drug Summit [82] to better inform politicians and the public about the various drug policy reform options [83]. Importantly, the involvement of people with lived experience in public education messaging will ensure that drug policy reform is more equitable and appropriately informed by those most affected by current policies [48].

Our findings clearly show most Australians support a health rather than a criminal response to drug possession, showing strong support for moving away from the criminalisation of illicit drugs. This is in stark contrast to estimated Australian Government expenditure on responses to drug use and related harms in 2021/22; the vast majority of expenditure was on law enforcement activities ($3.5B) with much smaller amounts spent on health (e.g., drug treatment) ($1.5B) and harm reduction ($89 M) responses [84]. More equitable funding for health responses and harm reduction initiatives that align with growing public support for these responses to illicit drugs is urgently needed.

Prioritising fair and accurate media reporting of harm reduction initiatives, illicit drugs and people who use drugs is pivotal to the implementation of evidence‐based drug policy reform [43, 48]. Inclusion of the voices of communities impacted most by drug policies (i.e., people who use drugs) in these narratives is also critical to ensure accurate representation [48]. AOD Media Watch undertake critical analysis of media reporting on alcohol and other drug related issues in Australia, to minimise misinformation [85]; this initiative is key to ensuring greater accountability in journalism and reducing undue media influence in the process of drug policy reform.

### 4.2 Limitations

Changes to the wording of survey questions and examples provided over time may impact the extent to which results can be compared. Notably, from 2007 onwards, the question measuring respondents' attitudes towards the legalisation of methamphetamine for personal use, provided ‘ice’ as an example. The significant negative media coverage surrounding the use of this term may have biassed responses towards opposition. Further, changes to demographics and survey modes offered over time may impact data comparability. Media searches were extensive but not exhaustive, hence some articles may have been missed, potentially resulting in the under‐representation of some topics. Analysis of jurisdictional changes in attitudes to drug policy over time would be beneficial given that implementation of drug policy reform occurs at state and territory levels at different times. Public opposition to the legalisation of illicit drugs for personal use may indicate a limitation in the way these questions are asked, without reference to the possible frameworks for legalisation [86]. While presentation of alternative policy options such as decriminalisation may be more palatable to the public than legalisation, the concept of decriminalisation can cause confusion among the general public as it may be equated to legalisation [87]. Our findings across several questions (including legalisation) about possible responses to possession of illicit drugs, however, highlighted the nuances in the spectrum of attitudes across these questions.

## 5 Conclusions

Australians are largely supportive of harm reduction initiatives while opposition towards the legalisation of most illicit drugs—particularly methamphetamine—remained high. Despite this, strong support for health‐based responses to drug possession highlights the nuanced nature of public attitudes towards illicit drugs. Media reporting during the period focused largely on methamphetamine, SIFs and decriminalisation/legalisation. Such coverage may shape public discourse in ways that do not reflect the evidence base. Fair and accurate media reporting of alcohol and other drug‐related issues should be prioritised to ensure more equitable and evidence‐based drug policy reform.

## Author Contributions

The first author designed the study methodology, analysed the data, and wrote and revised the manuscript. The last author conceived the projected, designed the study methodology, analysed the data and wrote and revised the manuscript.

## Funding

This work was supported by the National Health and Medical Research Council.

## Conflicts of Interest

The authors declare no conflicts of interest.

## Data Availability

The data that support the findings of this study are available from the Australian Institute of Health and Welfare. Restrictions apply to the availability of these data, which were used under licence for this study. Data are available from https://dataverse.ada.edu.au with the permission of the Australian Institute of Health and Welfare.

## Appendix A

**TABLE A1:** Changes in the questions assessing public support for harm reduction initiatives.

2001–2010	2013	2016–2022/23
Thinking now about the problems associated with heroin use, to what extent would you support or oppose measures such as…? Needle and syringe programs Regulated injecting rooms Trial of prescribed heroin	Thinking now about injecting drug use, to what extent would you support or oppose measures such as…? Some examples of injectable drugs are Heroin, Steroids, Speed, Ice and Pethidine. Needle and syringe programs Regulated injecting rooms Trial of prescribed heroin	Thinking now about injecting drug use, to what extent would you support or oppose measures such as…? Some examples of injectable drugs are Heroin, Speed and Ice. Needle and syringe programs Regulated injecting rooms Trial of prescribed heroin The availability of take‐home Naloxone, a drug that reverses the effects of a Heroin/methadone/morphine overdose Allowing potential drug users to test their pills/drugs at designated sites. The test will inform them of the purity and the substances the drug contains^a^

^a^ Only asked from 2019.

**TABLE A2:** Changes in the questions assessing public support for the legalisation of illicit drugs for personal use.

Still using the same scale and considering the following drugs, to what extent would you support or oppose the personal use of the following drugs being made legal…?		
2001	2004	2007–2022/23
Marijuana/Cannabis Heroin Amphetamines/speed Cocaine	Marijuana/Cannabis Heroin Methamphetamines/amphetamines (Speed) Cocaine	Marijuana/Cannabis Heroin Meth/amphetamines (i.e., Speed, Ice, Crystal, Base) Cocaine Ecstasy

**TABLE A3:** Changes in attitudes towards harm reduction initiatives over time—partial proportional odds ordinal regression models.

	Support vs. oppose		Ambivalent vs. oppose	
	OR (95% CI)	*p*	OR (95% CI)	*p*
SIF				
Year	0.96 (0.96, 0.97)	< 0.001	0.94 (0.95, 0.95)	< 0.001
Age	1.00 (1.00, 1.00)	< 0.01	1.01 (1.00, 1.01)	< 0.001
Sex				
Female	Ref.		Ref.	
Male	1.11 (1.08, 1.15)	< 0.001	1.16 (1.12, 1.20)	< 0.001
Recent illicit drug use				
No	Ref.		Ref.	
Yes	0.62 (0.59, 0.65)	< 0.001	0.64 (0.61, 0.68)	< 0.001
NSP				
Year	0.97 (0.97, 0.98)	< 0.001	0.96 (0.95, 0.96)	< 0.001
Age	1.00 (1.00, 1.01)	< 0.001	1.02 (1.01, 1.02)	< 0.001
Sex				
Female	Ref.		Ref.	
Male	1.27 (1.23, 1.32)	< 0.001	1.32 (1.27, 1.38)	0.001
Recent illicit drug use				
No	Ref.		Ref.	
Yes	0.62 (0.59, 0.65)	< 0.001	0.61 (0.57, 0.66)	< 0.001
Trial of prescribed heroin				
Year	0.98 (0.98, 0.98)	< 0.001	0.97 (0.97, 0.97)	< 0.001
Age	1.00 (0.99, 1.00)	< 0.001	1.00 (1.00, 1.00)	0.058
Sex				
Female	Ref.		Ref.	
Male	0.94 (0.90, 0.97)	0.001	0.99 (0.96, 1.03)	0.73
Recent illicit drug use				
No	Ref.		Ref.	
Yes	0.64 (0.61, 0.67)	< 0.001	0.67 (0.63, 0.70)	< 0.001
THN				
Year	0.96 (0.95, 0.97)	< 0.001	0.94 (0.93, 0.96)	< 0.001
Age	1.00 (1.00, 1.00)	0.99	1.01 (1.00, 1.01)	< 0.001
Sex				
Female	Ref.		Ref.	
Male	1.02 (0.96, 1.08)	0.58	0.99 (0.91, 1.07)	0.72
Recent illicit drug use				
No	Ref.		Ref.	
Yes	0.56 (0.52, 0.61)	< 0.001	0.55 (0.49, 0.62)	< 0.001

Abbreviations: CI, confidence interval; NSP, needle and syringe program; OR, odds ratio; SIF, supervised injecting facility; THN, take home naloxone.

**TABLE A4:** Changes in attitudes towards the legalisation of illicit drugs for personal use—partial proportional odds ordinal regression models.

	Support vs. oppose		Ambivalent vs. oppose	
	OR (95% CI)	*p*	OR (95% CI)	*p*
Cannabis				
Year	0.97 (0.97, 0.98)	< 0.001	0.97 (0.97, 0.97)	< 0.001
Age	1.01 (1.01, 1.01)	< 0.001	1.01 (1.01, 1.01)	< 0.001
Sex				
Female	Ref.		Ref.	
Male	0.85 (0.82, 0.88)	< 0.001	0.86 (0.83, 0.89)	< 0.001
Recent illicit drug use				
No	Ref.		Ref.	
Yes	0.11 (0.10, 0.12)	< 0.001	0.10 (0.09, 0.11)	< 0.001
Heroin				
Year	0.99 (0.98, 0.99)	< 0.001	0.98 (0.97, 0.98)	< 0.001
Age	1.00 (0.99, 1.00)	< 0.001	1.00 (1.00, 1.01)	< 0.001
Sex				
Female	Ref.		Ref.	
Male	0.77 (0.73, 0.81)	< 0.001	0.77 (0.73, 0.81)	< 0.001
Recent illicit drug use				
No	Ref.		Ref.	
Yes	0.07 (0.05, 0.10)	< 0.001	0.07 (0.05, 0.10)	< 0.001
Methamphetamine				
Year	0.99 (0.98, 0.99)	< 0.001	0.99 (0.98, 0.99)	< 0.001
Age	1.00 (1.00, 1.00)	0.55	1.01 (1.01, 1.01)	< 0.001
Sex				
Female	Ref.		Ref.	
Male	0.77 (0.73, 0.82)	< 0.001	0.77 (0.73, 0.82)	< 0.001
Recent illicit drug use				
No	Ref.		Ref.	
Yes	0.14 (0.13, 0.17)	< 0.001	0.14 (0.13, 0.17)	< 0.001
Cocaine				
Year	0.99 (0.98, 0.99)	< 0.001	0.99 (0.98, 0.99)	< 0.001
Age	1.00 (1.00, 1.00)	0.42	1.01 (1.01, 1.01)	< 0.001
Sex				
Female	Ref.		Ref.	
Male	0.71 (0.68, 0.75)	< 0.001	0.71 (0.68, 0.75)	< 0.001
Recent illicit drug use				
No	Ref.		Ref.	
Yes	0.19 (0.17, 0.22)	< 0.001	0.19 (0.17, 0.22)	< 0.001
Ecstasy				
Year	1.01 (1.00, 1.02)	0.05	1.00 (0.99, 1.01)	0.97
Age	1.00 (1.00, 1.01)	< 0.001	1.01 (1.01, 1.01)	< 0.001
Sex				
Female	Ref.		Ref.	
Male	0.68 (0.64, 0.71)	< 0.001	0.68 (0.64, 0.71)	< 0.001
Recent illicit drug use				
No	Ref.		Ref.	
Yes	0.14 (0.12, 0.16)	< 0.001	0.14 (0.12, 0.16)	< 0.001

Abbreviations: CI, confidence interval; OR, odds ratio.

**TABLE A5:** Changes in preferences for actions dealing with possession of illicit drugs (binary logistic regression).

Year	Health response	Civil response	Criminal response
	OR (95% CI)		
Cannabis			
2001	Ref.		
2022/23	1.64 (1.52, 1.77)***	0.59 (0.54, 0.64)***	0.81 (0.69, 0.94)**
Ecstasy			
2001	Ref.		
2022/23	1.43 (1.33, 1.53)***	0.93 (0.86, 1.00)	0.56 (0.52, 0.62)***
Heroin			
2001	Ref.		
2022/23	1.27 (1.18, 1.35)***	1.32 (1.22, 1.43)***	0.58 (0.54, 0.63)***
Methamphetamine			
2001	Ref.		
2022/23	1.23 (1.15, 1.32)***	0.84 (0.78, 0.91)***	0.88 (0.81, 0.95)**

*Note:* Age and sex controlled for **p* < 0.05; ***p* < 0.01; ****p* < 0.001.

Abbreviations: CI, confidence interval; OR, odds ratio.
